# Joint genotype inference with germline and somatic mutations

**DOI:** 10.1186/1471-2105-14-S5-S3

**Published:** 2013-04-10

**Authors:** Eric Bareke, Virginie Saillour, Jean-François Spinella, Ramon Vidal, Jasmine Healy, Daniel Sinnett, Miklós Csűrös

**Affiliations:** 1 Division of Hematology-Oncology, Sainte-Justine UHC Research Centre, Montréal, QC, Canada; 2 Department of Pediatrics, Faculty of Medicine, University of Montréal, QC, Canada; 3 Department of Computer Science and Operations Research, University of Montréal, QC, Canada

## Abstract

The joint sequencing of related genomes has become an important means to discover rare variants. Normal-tumor genome pairs are routinely sequenced together to find somatic mutations and their associations with different cancers. Parental and sibling genomes reveal *de novo *germline mutations and inheritance patterns related to Mendelian diseases.

Acute lymphoblastic leukemia (ALL) is the most common paediatric cancer and the leading cause of cancer-related death among children. With the aim of uncovering the full spectrum of germline and somatic genetic alterations in childhood ALL genomes, we conducted whole-exome re-sequencing on a unique cohort of over 120 exomes of childhood ALL quartets, each comprising a patient's tumor and matched-normal material, and DNA from both parents. We developed a general probabilistic model for such quartet sequencing reads mapped to the reference human genome. The model is used to infer joint genotypes at homologous loci across a normal-tumor genome pair and two parental genomes.

We describe the algorithms and data structures for genotype inference, model parameter training. We implemented the methods in an open-source software package (QUADGT) that uses the standard file formats of the 1000 Genomes Project. Our method's utility is illustrated on quartets from the ALL cohort.

## Background

Acute lymphoblastic leukemia (ALL) is the most common paediatric cancer and the leading cause of cancer-related death among children. Advances in the understanding of the pathobiology of ALL have led to risk-targeted treatment regimes and increased survival rates, but treatment is still far from optimal. Childhood ALL arises after the acquisition of a series of DNA sequence abnormalities. These initiating events, or so-called driver mutations, ultimately confer a selective growth advantage, and are causally implicated in cancer development. A central goal of cancer genome analysis is the identification of cancer genes that, by definition, carry driver mutations.

Next-generation sequencing (NGS) technologies [1] have enabled the genome-wide identification of human disease-related variants. Analysis pipelines have been established for the large-scale sequencing of individual tumor genomes [2]. Briefly, short sequencing reads are collected from the tumor sample, mapped to the reference genome assembly, and the set of aligned reads are used to infer variations across the genome at homologous loci covered with multiple reads. The sequence variants in the tumor genome may be the result of somatic mutations, or constitutional variants preserved in the somatic lineage. In order to distinguish somatic mutations from conserved variants, it is necessary to sequence normal and tumor samples side by side. The Cancer Genome Atlas Network [3] catalogues somatic mutations in different cancers using such normal-tumor pairs.

**Figure 1:**
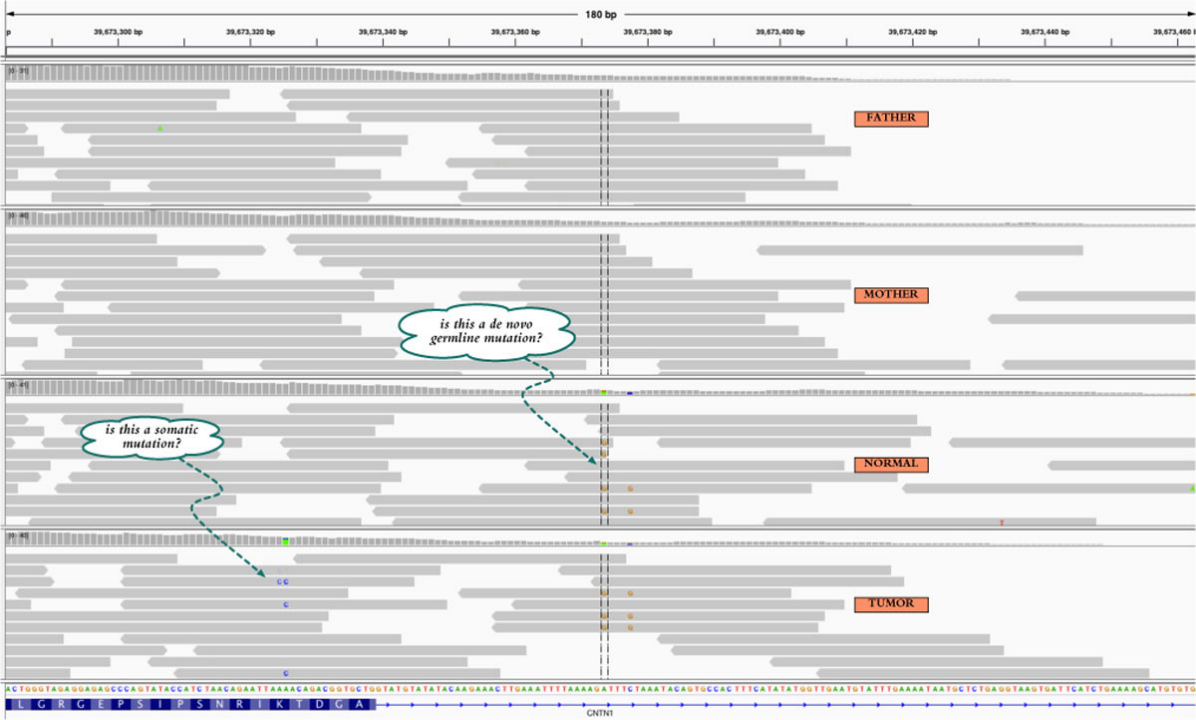
**Alignments for the normal, tumor, and parental reads samples highlight putative somatic and germline mutations**. The illustration shows a region along chromosome 12, displayed in the Integrative Genomics Viewer [7]. Mismatched bases are highlighted.

In general, genetic relationships (like normal-tumor pairs) can be efficiently exploited in genotype inference [4,5]. Inherited and *de novo *mutations can be traced through jointly sequenced family relatives [6]. Here, we consider variant detection in normal-tumor pairs coupled with parental samples. Such quartet data are used to categorize variants in the tumor and normal genomes by their origin: see Figure 1. One can readily classify inherited variants and *de novo *germline mutations by comparing the genotypes in the trio of normal and parental genomes. Likewise, somatic mutations correspond to differences between the normal and tumor genomes.

While sequencing an entire human genome is still too expensive for the average research laboratory, various target-enrichment techniques [8] are available for sequencing only regions of interest. In particular, sequencing the so-called *exome *covering all gene-coding regions, has been a routine step in medical applications [9]. Through our ongoing paediatric oncogenomics study, we conducted whole exome deep re-sequencing of a unique cohort of over 120 exomes of childhood ALL quartets, consisting of the patient's tumor and matched-normal material as well as DNA from both parents.

### Existing software tools

Various bioinformatics tools have been developed for genotyping individual genomes from sequencing data, including SNVMix [10], VarScan [11], and The Genome Analysis Toolkit GATK [12,13]. A couple of methods have been developed for the purpose of joint genotyping of paired normal-tumor samples, including SomaticSniper [14], MutationSeq [15], and JointSNVMix [16]. SomaticSniper and MutationSeq employ machine-learning techniques for variant classification; JointSNVMix is based on a full Bayesian model incorporating prior genotype distributions, somatic mutations, and sequencing base call errors. The Strelka software package [17] infers joint tumor-normal genotypes in a Bayesian model that also considers tumor sampling impurity: DNA collected from the tumor sample is usually "contaminated" to some degree with the normal tissue, and therefore the sequencing reads come from a mixture of normal and tumor genomes. To our knowledge, no existing variant caller incorporates somatic and germline mutation models simultaneously to handle quartet data as in our data sets.

### Our contribution

We infer the four genotypes jointly in a framework that respects the rules of inheritance in the germline and somatic lineages. Aside from assigning belief to *de novo *and somatic mutations, we hypothesized, constrained patterns in one lineage have an indirect beneficial effect on the inference in other lineages. In particular, the "triangulation" of the normal genome by related genomes means that genotypes and lineage-specific mutations can be resolved more reliably: information from the parental genotypes reinforce the inference of somatic mutations, and tumor sequencing reads help to recognize constitutional mutations. We present a Bayesian framework that incorporates prior parental genotypes, inherited, *de novo *and somatic mutations, as well as tumor-sampling impurity and sequencing errors. All model parameters are estimated in an expectation-maximization algorithm [18].

## Methods

### Probabilistic model

**Figure 2:**
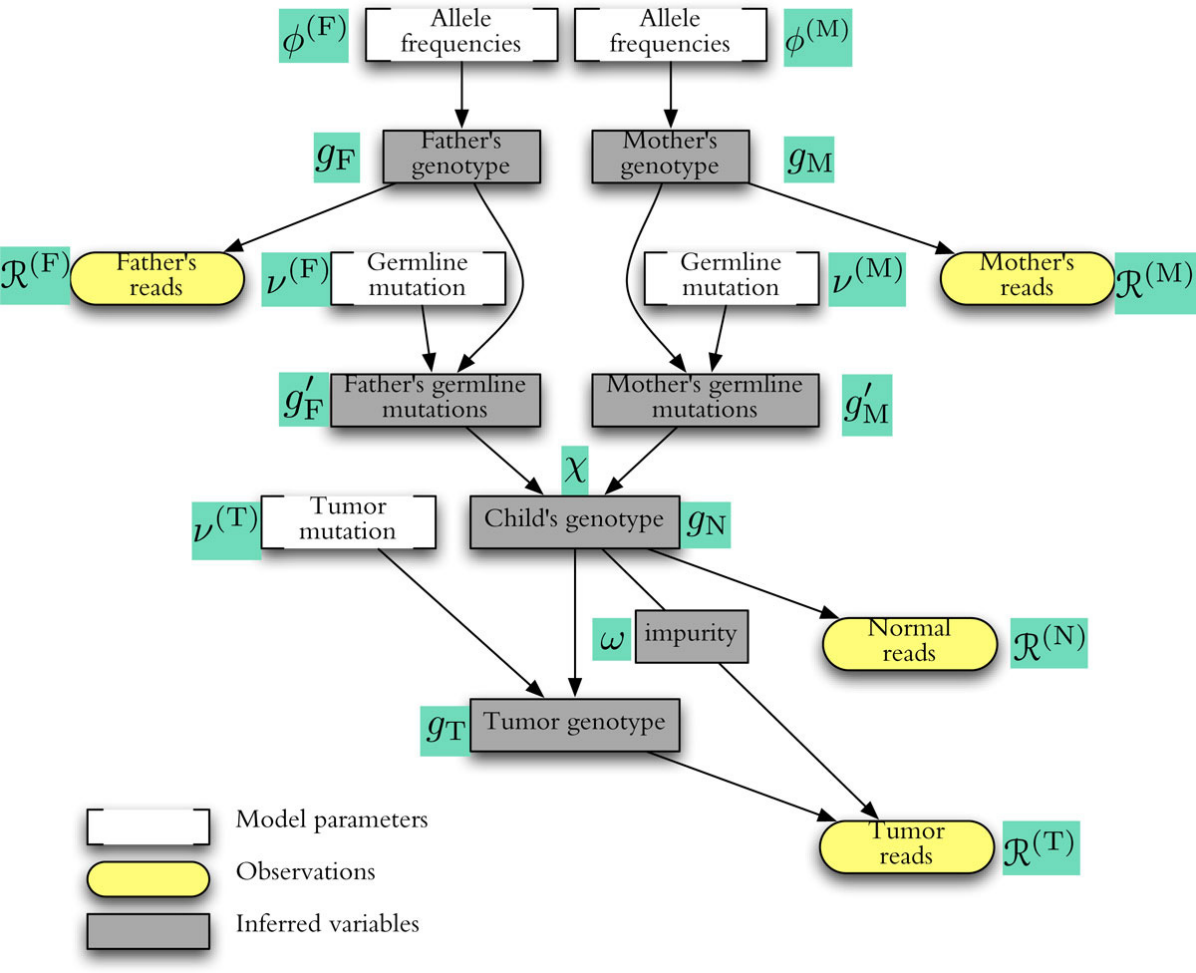
**Probabilistic model for base calls covering a single locus, with dependencies among genotypes and sequencing reads**. See Equation (5) for the corresponding likelihood formula.

Figure 2 illustrates our model of single-nucleotide polymorphisms at homologous loci across four genomes linked by inheritance and somatic mutations. The model quantifies the descent-by-modification relationships between the unknown genotypes via three sets of parameters. First, a genotype frequency model is assumed for the parental genotypes. Second, we assume a standard DNA substitution model for the frequency of germline mutations. The parental diploid genotypes determine the child's normal genotype by Mendelian inheritance. (For simplicity, we discuss only diploid genotypes: our implementation considers sex chromosomes in an analogous manner, but using the appropriate inheritance model.) Finally, another DNA substitution model with its own parameters determines mutations in the tumor genome.

Base calls are assumed to be independent between different loci. The input for genotype inference at a single locus consists of nucleotide base calls made with their accompanying sequencing error probabilities.

#### Parental genotype priors

Let *π*[*x*] denote the frequency of each allele *x *∈ {A; C; G; T} at a given locus. The prior allele frequencies are computed by using a standard DNA substitution model quantifying the divergence from the reference genome assembly. Assuming the simple Jukes-Cantor model with a reference nucleotide genotype y,π[x]=ν/3 for x≠y and $\Pi \left[y\right]=1-v$, where *v *is the parental genome's divergence from the reference. More general divergence models and known SNP frequencies can be accommodated by using $\pi \left[x\right]=\sum_{y\in \left\{\text{A},\text{C},\text{G},\text{T}\right\}}\pi_{\text{ref}}\left[y\right]v_{y\to x}$, where $V_{x\to y}$ is the model's substitution probability from x  to y  and $\Pi_{\text{ref}}$ is a known allele frequency.

For a diploid locus, let $\phi \left(xx\right)\;\text{and}\;\phi \left(xy\right)$ denote the frequency of homozygous xx and heterozygous xy genotypes. Allele frequencies immediately determine diploid allele frequencies in standard Hardy-Weinberg equilibrium: $\phi \left(xx\right)={\left(\pi \left[x\right]\right)}^{2}$ for a homozygous locus xx, and ϕ(xy)=2π[x]π[y] for a heterozygous xy. In order to accommodate different homozygous-heterozygous ratios, we employ an additional parameter γ≤1.

(1) $$\begin{array}{c} \phi \left(xx\right)=\left(1+\gamma \frac{1-\pi \left[x\right]}{\pi \left[x\right]}\right)\;\cdot \;{\left(\pi \left[x\right]\right)}^{2}\;\left(\text{homozygous}\;xx\right)\\ \phi \left(xy\right)=2\left(1-\gamma \right)\;\cdot \;\pi \left[x\right]\;\cdot \;\pi \left[y\right]\;\left(\text{hetrozygous}\;xy\right) \end{array}$$

(In other words, *γ *is numerically analogous to the so-called inbreeding coefficient, or F-statistic.) It is easy to verify that haploid allele frequencies are the same at any γ setting.

#### Mutations and inheritance

The child's normal genotype is determined by Mendelian inheritance and *de novo *point mutations with probabilities *v_x_*_→*y *_that occur within the parental germlines. For simplicity, germline mutations in a parental lineage (*g*_F, _*g*_M _for father and mother, respectively) are conceived of as mutations that result in a diploid genotype$\left(g_{\text{F}}^{\prime },g_{\text{M}}^{\prime }\right)$, which then determine the child's normal genotype by Mendel's laws.

Germline mutations follow standard molecular evolution model for substitutions in DNA. Let *X *denote the parent's normal allele at a locus, and *X' *denote the same allele at the end of the germline before gametogenesis. The mutation model specifies the probabilities that apply to every locus $v_{x\to x^{\prime }}=\mathbb{P}\left\{X^{\prime }=x|X=x\right\}$. Let $\chi \left(g_{\text{F}}^{\prime },g_{\text{M}}^{\prime }\to g_{\text{N}}\right)$ denote the probability of normal genotype *g*_N _given the mutated parental genotypes $g_{\text{F}}^{\prime },g_{\text{M}}^{\prime }$. Then χ may be 0, 1, 1/2 or 1/4, depending on the common alleles between the three genotypes.

##### Tumor genotype

The tumor genome undergoes mutations following the same type of molecular evolution model as the one used for germline mutations, but has its own parameters.

#### Sequencing errors

The alignment at the locus is represented as a set of basecall-error probability pairs (*z_k_*, *∈_k_*) for *k *= 1, ..., *m*. The *k*-th read calls allele *z_k _*with an error probability of 0 <*∈_k _*≤ 1. Typically, the aligned sequencing reads give nucleotide base calls and accompanying error probabilities on a logarithmic integer scale [19,20], called the Phred scale. (In principle, the input SAM- or BAM-format file gives the Sanger-encoded sequencing error in the QUAL column or the OQ tag.) Namely,

$$\varepsilon_{k}=\mathbb{P}\left\{\text{sample}\;\text{allele}\;\text{is}\;\text{different}\;\text{from}\;z_{k}|\text{sequencer outputs}\;z_{k}\right\}=10^{-q_{k}/10}=\phi^{q_{k}},$$

where *q_k _*is the reported quality score, and $\phi =\sqrt[{10}]{1/10}=0.794\cdots$. Let *Z *denote the base call, and *Y *denote the true nucleotide. We assume that errors are unbiased in the sense that

$$\mathbb{P}\left\{Z=z|Y=y\right\}=\left\{\begin{array}{cc} 1-\varepsilon & \left\{y=z\right\}\\ \varepsilon /3 & \left\{y\neq z\right\} \end{array}\right)$$

#### Allele sampling and sample impurity

Aligned sequencing reads randomly sample the haploid alleles at a given locus. Let *y_k _*be the true allele for base call *z_k_*. The locus' diploid genotype determines the frequency $\rho \left[y\right]=\mathbb{P}\left\{y_{k}=y\right\}$ for each possible allele *y*. At a homozygous locus *xx*, *ρ*[*y*] = 1 and *ρ*[*x'*] = 0 for all *x' *≠ *x*. At a heterozygous locus *xx'*, *ρ*[*y*] = 1/2, *ρ*[*x'*] = 1/2 and *ρ*[*x"*] = 0 for all *x" *≠ *x*, *x'*.

Impure tumor samples have a mixed distribution, which is the linear combination of the normal and tumor genotype distributions. For tumor reads, *ρ*[*y*] comes from a mixture (0 ≤ *ω *≤ 1; *ω *= 1 for pure tumor sampling) between the normal and tumor genotypes:

(3) $$\rho \left[y\right]=\rho_{\text{N}}\left[y\right]\;\;\cdot \;\;\left(1-\omega \right)+\rho_{\text{T}}\left[y\right]\;\;\cdot \;\;\omega .$$

#### Likelihood for aligned reads given the genotypes

Suppose we are given the set of basecall-error probability pairs (*z_k_*, *∈_k_*) for *k *= 1, ..., *m*, representing the alignment at a locus. Let *Z_k _*be the random variable for base call in read *k *at a fixed error rate *∈_k_*, and let *Y_k _*be the random variable for the true sampled allele. Define the base call probability

$$p_{k}\left(y\right)\;=\;\mathbb{P}\left\{Z_{k}\;=\;z_{k}|Y_{k}\;=\;y\right\}\;=\;\left\{\begin{array}{cc} 1-\varepsilon_{k} & \left\{y=z_{k}\right\}\\ \varepsilon_{k}/3 & \left\{y\neq z_{k}\right\} \end{array}\right)$$

Hence, $\mathbb{P}\left\{Z_{k}=z_{k}\right\}=\sum_{y}\mathbb{P}\left\{Z_{k}=z_{k}|Y_{k}=y\right\}\mathbb{P}\left\{Y_{k}=y\right\}=\sum_{y}p_{k}\left(y\right)\rho \left[y\right]$.

The read likelihood for a given allele distribution *ρ *is defined as

$$L\left(\rho \right)=p\left(\left(z_{k},\;\varepsilon_{k}\right):k=1,\;\ldots ,\;m\right)=\mathbb{P}\left\{\forall k=1,\;\ldots ,\;m:Z_{k}=z_{k}\right\}$$

Since base calls are independent across reads when conditioned on the allele mixture in the sample,

(4) $$L(\rho )=\underset{k=1,\;\ldots ,m}{\prod^{\text{​}}}\mathbb{P}\{Z_{k}=z\}=\underset{k}{\prod^{\text{​}}}\underset{\text{probability for read }k}{\underset{︸}{\underset{y}{\sum^{\text{​}}}p_{k}(y)\rho [y]}}$$

#### Complete likelihood

Let $g_{\text{F}}$, $g_{\text{M}}$, $g_{\text{N}}$, $g_{\text{T}}$denote the diploid genotypes for father, mother, normal, and tumor samples, respectively. Let $g_{\text{F}}^{\prime }$ and $g_{\text{M}}^{\prime }$ denote the parental genotypes after germline mutations. These six random variables constitute the hidden variables in our probabilistic model. The input is a set of aligned sequenced bases from each of the four samples:$R^{\left(\text{F}\right)}$, $R^{\left(\text{M}\right)}$, $R^{\left(\text{N}\right)}$, $R^{\left(\text{T}\right)}$. Every set R  consists of base call and error pairs (*z_k_*, *∈_k_*). The likelihood for the aligned base calls is then

(5) $$L=\underset{g_{\text{F}},g_{\text{M}},{g^{\prime }}_{\text{F}},{g^{\prime }}_{\text{M}},g_{\text{N}},g_{\text{T}}}{\sum }\underset{=\;L\;\left(g_{\text{F}},g_{\text{M}},{g^{\prime }}_{\text{F}},{g^{\prime }}_{\text{M}},g_{\text{N}},g_{\text{T}}\right)}{\underset{︸}{\mathbb{P}\left\{R^{\text{(F)}},R^{\text{(M)}},R^{\text{(N)}},R^{\text{(T)}}|g_{\text{F}},g_{\text{M}},{g^{\prime }}_{\text{F}},{g^{\prime }}_{\text{M}},g_{\text{N}},g_{\text{T}}\right\}}}\times \underset{=\;p\;\left(g_{\text{F}},g_{\text{M}},{g^{\prime }}_{\text{F}},{g^{\prime }}_{\text{M}},g_{\text{N}},g_{\text{T}}\right)}{\underset{︸}{\mathbb{P}\left\{g_{\text{F}},g_{\text{M}},{g^{\prime }}_{\text{F}},{g^{\prime }}_{\text{M}},g_{\text{N}},g_{\text{T}}\right\}}}$$

The $L\left(g_{\text{F}},...\right)$ factor is the likelihood for the reads, given the genotypes. By the independence of the sequencing runs,

(6) $$L\left(g_{\text{F}},g_{\text{M}},g_{\text{F}}^{\prime },g_{\text{M}}^{\prime },g_{\text{N}},g_{\text{T}}\right)=\underset{\text{father's}\;\text{reads}}{\underset{︸}{L^{\left(\text{F}\right)}\left(g_{\text{F}}\right)}}\times \underset{\text{mother's}\;\text{reads}}{\underset{︸}{L^{\left(\text{M}\right)}\left(g_{\text{M}}\right)}}\times \underset{\text{normal}\;\text{reads}}{\underset{︸}{L^{\left(\text{N}\right)}\left(g_{\text{N}}\right)}}\times \underset{\text{impure}\;\text{tumor}\;\text{reads}}{\underset{︸}{L^{\left(\text{T}\right)}\left(g_{\text{N}},g_{\text{T}}\right)}}$$

The four factors are defined by (4), via the allele frequencies *ρ *that are determined by genotypes and tumor sampling purity, as discussed above (see **Allele sampling and sample impurity**).

The $p\left(g_{\text{F}},...\right)$ factor in (5) covers all mutation and inheritance events, as well as the parental genotypes. By the dependencies depicted in Figure 2,

(7) $$\begin{array}{ccc} p\left(g_{F},g_{M},{g^{\prime }}_{F},{g^{\prime }}_{M},g_{N},g_{T}\right)= & \phi^{\left(F\right)}\left(g_{F}\right)\times \phi^{\left(M\right)}\left(g_{M}\right) & \left(\text{parental}\;\text{genotype}\;\text{priors}\right)\\ & \times \nu^{\left(F\right)}\left(g_{F}\to {g^{\prime }}_{F}\right)\;\times \;\nu^{\left(M\right)}\left(g_{M}\to {g^{\prime }}_{M}\right) & \left(\text{germline}\;\text{mutations}\right)\\ & \times \chi \left({g^{\prime }}_{F},\;{g^{\prime }}_{M}\to g_{N}\right) & \left(\text{inheritance}\right)\\ & \times \nu^{\left(T\right)}\left(g_{N}\to g_{T}\right) & \left(\text{tumor}\;\text{mutations}\right) \end{array}$$

### Algorithmic techniques and data structures

Our algorithmic solutions address the efficient calculation of the likelihood formula of (5), and its use in an Expectation-Maximization (EM) framework for model parameter setting. First, we examine a straightforward decomposition of the likelihood formula dictated by the assumed probabilistic graphical model.

For the EM algorithm, we need to recompute likelihoods and posterior probabilities in a number of iterations, which can be directly achieved by storing all sequencing reads in memory, but such an approach may be costly. We scrutinize the computation of read likelihoods, in order to arrive at an economical data structure, also discussed in some detail, that eliminates the need to store all base calls in memory.

#### Likelihood decomposition

The summation formula for the full likelihood in Equation (5) is rearranged for efficiency, using the independencies apparent in (6) and (7). In addition, the germline mutations can be combined with Mendelian inheritance: define $\chi^{\prime }\left(g_{\text{F}},g_{\text{M}}\to g_{\text{N}}\right)$ as

$$\chi^{\prime }\left(g_{\text{F}},\;g_{\text{M}}\to g_{\text{N}}\right)=\underset{{g^{\prime }}_{\text{F}},{g^{\prime }}_{\text{M}}}{\sum }\nu^{\left(\text{F}\right)}\left(g_{\text{F}}\to g_{\text{F}}^{\prime }\right)\;\text{. }\nu^{\left(\text{M}\right)}\left(g_{\text{M}}\to g_{\text{M}}^{\prime }\right)\;\text{. }\chi \left(g_{\text{F}}^{\prime },\;g_{\text{M}}^{\prime }\to g_{\text{N}}\right).$$

Then

(8) $$\begin{array}{c} L=\underset{g_{\text{F}}}{\sum }\phi^{\left(\text{F}\right)}\left(g_{\text{F}}\right)\cdot L^{\left(\text{F}\right)}\left(g_{\text{F}}\right)\times \underset{g_{\text{M}}}{\sum }\phi^{\left(\text{M}\right)}\left(g_{\text{M}}\right)\cdot L^{\left(\text{M}\right)}\left(g_{\text{M}}\right)\\ \;\times \underset{g_{\text{N}}}{\sum }\chi^{\prime }\left(g_{\text{F}},\;g_{\text{M}}\to g_{\text{N}}\right)\;\text{. }L^{\left(\text{N}\right)}\left(g_{\text{N}}\right)\times \underset{g_{\text{T}}}{\sum }\nu^{\left(\text{T}\right)}\left(g_{\text{N}}\to g_{\text{T}}\right)\;\text{. }L^{\left(\text{T}\right)}\left(g_{\text{N}},\;g_{\text{T}}\right). \end{array}$$

If there are *G *possible diploid genotypes (*G *= 10 for DNA with four alleles), Equation (8) shows that the likelihood can be computed in *O*(*G*^3^) time, instead of *O*(*G*^6^) suggested by the definition of (5). In particular, the likelihood computation proceeds by calculating the following values.

$$\begin{array}{c} {\text{L}}_{\text{N}}[g]=L^{\left(\text{N}\right)}(g)\times \sum_{g_{\text{T}}}\nu^{\left(\text{T}\right)}(g\to g_{\text{T}})\cdot L^{\left(\text{T}\right)}(g,\;g_{\text{T}})\\ {\text{L}}_{\text{FM}}[g,\;g^{\prime }]=\sum_{g_{\text{N}}}L^{\left(\text{F}\right)}(g_{\text{F}})\;\cdot \;L^{\left(\text{M}\right)}(g_{\text{M}})\;\cdot \;\chi^{\prime }(g,\;g^{\prime }\to g_{\text{N}})\;\cdot \;{\text{L}}_{\text{N}}[g_{\text{N}]}]\\ L=\sum_{g,g^{\prime }}\phi^{\left(\text{F}\right)}(g)\;\text{​}\cdot \;\phi^{\left(\text{M}\right)}(g^{\prime })\;\cdot \;{\text{L}}_{\text{FM}}[g,\;g^{\prime }] \end{array}$$

#### Read likelihoods

Equation (4) is conveniently rearranged by different base calls:

(9) $$L\left(\rho \right)=\underset{=L_{z}\left(\rho \right)}{\underset{︸}{\underset{z}{\prod }\underset{k:\;\;z_{k}=z}{\prod }\underset{\text{probability for read }k}{\underset{︸}{\left(\underset{y}{\sum }p_{k}\left(y\right)\rho \left[y\right]\right)}}}}.$$

Each subproduct is calculated separately as

(10) $$L_{z}\left(\rho \right)=\underset{k:\;\;z_{k}=z}{\prod }\left(\underset{y}{\sum }p_{k}\left(y\right)\rho \left[y\right]\right)=\underset{k:\;\;z_{k}=z}{\prod }\left(p_{k}\left(z\right)\rho \left[z\right]+\underset{y\neq z}{\sum }p_{k}\left(y\right)\rho \left[y\right]\right)\;=\underset{k:\;\;z_{k}=z}{\prod }\left(\rho \left[z\right]+\varepsilon_{k}\frac{1-4\rho \left[z\right]}{3}\right),$$

by Equation (2). Note that only the called base's frequency *β *= *ρ*[*z*] appears in the formula. Define

$$f\left(\beta ,\;\varepsilon \right)=\beta +\varepsilon \frac{1-4\beta }{3},\;\text{and}\;T_{\beta }\left[z\right]=\underset{k:\;z_{k}=z}{\prod }f\left(\beta ,\;\varepsilon_{k}\right).$$

If no reads call *z*, then *T_β_*[*z*] = 1. Equation (10) becomes *L_z_*(*ρ*) = *T_ρ_*_[*z*]_[*z*]. For pure diploid samples, *ρ*[*z*] may be 0, 1 or 1/2, corresponding to the possible subproducts for diploid samples *E*[*y*] = *T*_0_[*y*] (sequencing error), *C*[*y*] = *T*_1_[*y*] (homozygote *yy*), and *H*[*y*] = *T*_1/2_[*y*] (heterozygote with *y*). Likelihood formulas become even more economical with normalized subproducts *C'*[*y*] = *C*[*y*]/*E*[*y*], *H'*[*y*] = *H*[*y*]/*E*[*y*], and scaling factor $E=\prod_{y}E\left[y\right]=\prod_{k}\frac{1}{3}\varepsilon_{k}.$

**Homozygous sample**. For a pure homozygous sample with genotype *yy *(*ρ*[*y*] = 1 and *ρ*[*z*] = 0 for *z *≠ *y*),

(11) $$L\left(yy\right)=C\left[y\right]\times \underset{z\neq y}{\prod }E\left[z\right]=C^{\prime }\left[y\right]\times E$$

**Heterozygous sample**. For a pure heterozygous sample *yy' *(*y *≠ *y'*; *ρ*[*y*] = *ρ*[*y'*] = 1/2 and *ρ*[*z*] = 0 for *z *≠ *y*, *y'*), the likelihood becomes

(12) $$L\left(yy^{\prime }\right)=\underset{z=y,y^{\prime }}{\prod }H\left[z\right]\times \underset{z\neq y,y^{\prime }}{\prod }E\left[z\right]=H^{\prime }\left[y\right]\times H^{\prime }\left[y^{\prime }\right]\times E$$

**Figure 3:**
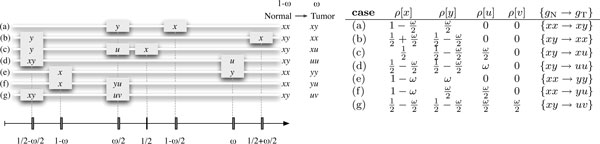
**Allele frequencies with impure normal-tumor genotypes that differ from each other**. Reads sample the tumor with probability *ω*, and the normal genotype with 1 - *ω*.

**Impure tumor sample**. The pure tumor $\left(g_{\text{T}}\right)$ and normal genotypes $\left(g_{\text{N}}\right)$ are proper diploid genotypes. Tumor sequencing reads come from an impure sample: they sample the tumor genotype with probability *ω*, and the normal genotype with probability (1 - *ω*). By Eq. (3), identical genotypes correspond to identical allele frequencies *ρ*, no matter what the purity level *ω *is. Suppose, however, that the locus has a mixture of *divergent *normal and tumor genotypes. Figure 3 shows that there are up to four correct base calls appearing in the reads, depending on the tumor mutation pattern $g_{\text{N}}\to g_{\text{T}}$. There are 6 possible queried allele frequencies *β *≠ 0, 1, 1/2 (Figure 3):

(13) $$\begin{array}{l} \underline{\;\;\;\;\beta =\rho [z]\;\;\;\;f(\beta ,\varepsilon )\;\;\;\;}\;\;\;\underline{\;\;\;\;\beta =\rho [z]\;\;\;\;f(\beta ,\varepsilon )\;\;\;\;\;}\\ \;\underline{\begin{array}{ll} \;\frac{1}{2}-\frac{\omega }{2} & \;\frac{1}{2}-\frac{1}{2}\omega +\varepsilon \left(\frac{2}{3}\omega -\frac{1}{3}\right)\\ \;1-\omega & \;\;\;1-\omega +\varepsilon (\frac{4}{3}\omega -1)\\ \;\frac{\omega }{2} & \;\;\;\frac{1}{2}\omega -\varepsilon \left(\frac{2}{3}\omega -\frac{1}{3}\right) \end{array}}\;\;\;\;\underline{\begin{array}{ll} \;1-\frac{\omega }{2} & \;1-\frac{1}{2}\omega +\varepsilon \left(\frac{2}{3}\omega -1\right)\\ \;\omega & \;\;\;\;\;\omega -\varepsilon (\frac{4}{3}\omega -\frac{1}{3})\\ \;\frac{1}{2}+\frac{\omega }{2} & \;\frac{1}{2}+\frac{1}{2}\omega -\varepsilon \left(\frac{2}{3}\omega +\frac{1}{3}\right)\; \end{array}} \end{array}$$

If the normal and tumor genotypes are identical, then the purity is immaterial. The formulas with somatic mutations are:

(14a) $$L\left(xx,\;xy\right)=T_{1-\frac{\omega }{2}}^{\prime }\left[x\right]\;\times \;T_{\frac{\omega }{2}}^{\prime }\left[y\right]\;\times \;E\;\left\{xx\to xy\right\}$$

(14b) $$L\left(xy,\;xx\right)=T_{\frac{1}{2}+\frac{\omega }{2}}^{\prime }\left[x\right]\;\times \;T_{\frac{1}{2}-\frac{\omega }{2}}^{\prime }\left[y\right]\times E\;\left\{xy\to xx\right\}$$

(14c) $$L\left(xy,\;xu\right)=H^{\prime }\left[x\right]\times T_{\frac{1}{2}-\frac{\omega }{2}}^{\prime }\left[y\right]\;\times \;T_{\frac{\omega }{2}}^{\prime }\left[u\right]\;\times \;E\;\left\{xy\to xu\right\}$$

(14d) $$L\left(xy,\;uu\right)=T_{\frac{1}{2}-\frac{\omega }{2}}^{\prime }\left[x\right]\times T_{\frac{1}{2}-\frac{\omega }{2}}^{\prime }\left[y\right]\times T_{\omega }^{\prime }\left[u\right]\times E\;\left\{xy\to uu\right\}$$

(14e) $$L\left(xx,\;yy\right)=T_{1-\omega }^{\prime }\left[x\right]\;\times \;T_{\omega }^{\prime }\left[y\right]\times E\;\left\{xx\to yy\right\}$$

(14f) $$L\left(xx,\;yu\right)=T_{1-\omega }^{\prime }\left[x\right]\times T_{\omega /2}^{\prime }\left[y\right]\times T_{\omega /2}^{\prime }\left[u\right]\;\times \;E\;\left\{xx\to yu\right\}$$

(14g) $$L\left(xy,\;uv\right)=T_{\frac{1}{2}-\frac{\omega }{2}}^{\prime }\left[x\right]\times T_{\frac{1}{2}-\frac{\omega }{2}}^{\prime }\left[y\right]\times T_{\frac{1}{2}+\frac{\omega }{2}}^{\prime }\left[u\right]\times T_{\frac{1}{2}+\frac{\omega }{2}}^{\prime }\left[v\right]\times E\;\left\{xy\to uv\right\}$$

#### Data structure for storing base calls

Storing individual base calls at each locus is costly, because the sequencing coverage may be large across the four samples. It suffices, however, to store the partial likelihood factors appearing in Equations (11), (12) and (14). In particular, for each locus with mapped base calls, it is enough to store the sample-specific *H'*[*x*], *C'*[*x*], and $T_{\beta }^{\prime }\left[x\right]$ values for each called base *x *and the six possible values of *β*, in addition to a single scaling value *E*. For a sample with *m *base calls at the locus, (1 + 8*m*) variables thus suffice, independently of read coverage. The stored partial likelihoods are reused throughout the iterations optimizing the model parameters at a fixed tumor purity level *ω*.

##### Recurrent base calls

In our experience, loci with identical sets of base calls reoccur at an appreciable frequency, especially at lower coverages (less than about 15×) that characterize exon boundaries in exome sequencing. We exploit recurrent patterns in the piled-up base calls to achieve even better memory usage and speed. Namely, we sort the base calls R  at a given locus for a given sample (by allele and quality score), and use run-length encoding to achieve a compact characterization h(R). The encoding is not used for higher coverages or widely varying quality scores, where *h *would take too many bits. Information-theoretic considerations [21] suggest that compactly encoded R  occur more often ( h is our proxy for Kolmogorov complexity). Short codes  h are placed in a small hash table to find recurrent calls (in our experiments with 20-30× total coverage by AB SOLiD sequencing reads, 20-30% savings can be achieved this way).

#### Parameter optimization and genotype inference

The genotypes, germline and tumor mutations are inferred by carrying out the summation of (5) for a restricted set of genotypes in order to calculate posterior probabilities. For example, in order to infer the child's normal genotype, calculate first

$${\text{U}}_{\text{N}}\left[g\right]=\underset{g_{\text{F}},g_{\text{M}}}{\sum }\phi^{\left(\text{F}\right)}\left(g_{\text{F}}\right)\cdot L^{\left(\text{F}\right)}\left(g_{\text{F}}\right)\cdot \phi^{\left(\text{M}\right)}\left(g_{\text{M}}\right)\cdot L^{\left(\text{M}\right)}\left(g_{\text{M}}\right)\cdot \chi^{\prime }\left(g_{\text{F}},\;g_{\text{M}}\to g\right).$$

Then the child's normal genotype has posterior probabilities $p_{\text{N}}\left[g\right]=\frac{{\text{U}}_{\text{N}}\left[g\right]\cdot {\text{L}}_{\text{N}}\left[g\right]}{L}$.

Model parameters are optimized using the EM algorithm [18]. In one iteration, likelihoods and various posterior probabilities are computed across all loci in the so-called E-step, which are then used to set the model parameters for the next iteration in the so-called M-step. The iterations continue until convergence is achieved. Among the optimized model parameters, the parental genotype priors, the germline and tumor mutation parameters are optimized through multiple iterations using the same set of precalculated partial likelihoods.

Setting the single parameter of the Jukes-Cantor model for the germline and tumor mutations is fairly straightforward by using posterior probabilities. For example, the tumor mutation parameter $\nu^{\left(\text{T}\right)}$ is set by summing the posterior probabilities for allele substitutions across all loci:

(15) $$\hat{\nu }=\frac{\sum_{j=1}^{N}\sum_{g_{\text{N}},g_{\text{T}}}\alpha \left(g_{\text{N}},g_{\text{T}}\right)\;\cdot \;p_{j}\left(g_{\text{N}},g_{\text{T}}\right)}{2N},$$

where *N *is the number of loci, $p_{j}\left(g_{\text{N}},\;g_{\text{T}}\right)$ is the posterior probability for a normal-tumor genotype pair at locus *j*, and *α*(*g*, *g'*) is the expected number of substitutions for the two alleles given the diploid genotypes.

In order to set the tumor purity *ω*, the partial likelihoods need to be recomputed (for different *T_β _*values) by reading the input read-mapping files at each iteration. At the same time, we compute a calibrated map μ:{0,1,…,93}↦[0,1] from reported base-calling qualities to sequencing errors in the same framework. Note that both *μ *and *ω *have well-estimated initial values (*μ *starts with the canonical Phred-scaled values, and *ω *is estimated experimentally).

##### Decomposing zygosity and divergence

For the purposes of parameter inference, consider the following machine realizing the formulas for the parental genotype priors of (1). Upon receiving a heterozygous genotype *xy*, it flips either allele to output homozygous *xx *or *yy *with *γ */2 probability each. Otherwise, with probability (1 - *γ*), the output is the same heterozygote *xy*. Homozygous genotypes are output without any change. Clearly, if the input genotype distribution is for Hardy-Weinberg, then the machine's output is distributed by the probabilities of (1). Accordingly, the divergence and heterozygosity parameters for the parental genotype prior *ϕ *are inferred by treating the machine's input genotype as a hidden variable. Expected frequencies for divergent input genotypes are used to estimate the divergence parameter, and expected frequencies for heterozygous → homozygous "flips" are used to estimate *γ*.

### Sequencing data

#### Exome sequencing

We conducted validation experiments using exome-sequencing reads for two sets of quartets (**A **and **B**) generated on the Child Health Genomics Platform of the Sainte-Justine UHC Research Center. Sequencing reads were produced on Applied Biosystem's SOLiD sequencer and mapped with the accompanying software. Table 1 summarizes statistics on the mapped sequencing reads.

**Table 1:** Coverage statistics for two quartets used in validation experiments.

Exome sequencing (AB SOLiD system)					
	**Total**	**Normal**	**Tumor**	**Father**	**Mother**

**Quartet A **(chromosome 12 only)					
Sites:	11 458 426				
Reads:	5 530 702	1 454 529	1 396 361	1 117 647	1 562 165
Depth:	23.0	6.0	5.8	4.6	6.6
Experimental tumor purity:			0.63		
QUADGT's purity estimate:			0.41		

**Quartet B **(all chromosomes)					
Sites:	425 344 130				
Reads:	134 574 732	38 156 404	34 580 382	29 997 426	31 840 520
Depth:	23.0	5.4	5.1	6.5	5.9
Experimental tumor purity:			0.97		
QUADGT's purity estimate:			0.44		

**Whole-genome sequencing **(Illumina HiSeq 2000)					
**Quartet B **(whole genome)					
		
	**Total**	**Normal**	**Tumor**		
		
Depth:	123.8	48.6	75.2		
Illumina's purity estimate:			0.46		

#### Whole-genome sequencing

Tumor and normal DNA samples from Quartet B were submitted to Illumina, Inc, for deep whole-genome sequencing using the standard operating procedures of the HiSeq 2000 sequencing platform. Table 1 summarizes coverage statistics and tumor impurity. The whole-genome data was further analyzed for somatic mutations with CASAVA and Strelka [17] by Illumina, Inc.

### Implementation

We incorporated the presented methods into an open-source Java software package called QUADGT, using the standard file formats of the 1000 Genomes Project (SAM v1.4 [20] for input and VCF v4.1 [22] for output). Any of the input files may be missing, which makes QUADGT suitable to analyze sets with just normal-tumor samples, or just parental-offspring trios.

The probabilistic framework enabled us to couple the inference with confidence measures in the form of quality scores computed from posterior probabilities. The quality scores accompany sample-specific genotype calls (VCF's GQ field), as well as the joint genotyping for the four samples (VCF's QUAL column). The posterior probabilities for germline and somatic mutations are calculated, as well, by summing across all pertinent genotype assignments. The program specifically introduces ambiguity in the genotyping calls to meet prescribed quality scores: a definite *x*/*y *base call in a sample is replaced by *x/*. or ./*y*, and then by ./. in a greedy manner, in order to achieve high specificity.

#### Parallelization

The E-step of the optimization, where sums of posterior probabilities are calculated across loci, is well-suited to parallelization. In our implementation, we use a hash key computed at each locus to assign the calculations to different computing threads running in parallel.

#### Availability

The QUADGT software package is publicly available at http://www.iro.umontreal.ca/~csuros/quadgt/.

## Results and discussion

We used two quartet data sets (**A **and **B**) to compare independent and joint variant detection. Figure 1 summarizes the coverage statistics for the quartets. Exome-sequencing reads at 5-6× coverage per sample were mapped to the human reference, and we inferred genome variants using our software package QUADGT. The entire analysis pipeline for one quartet set, including model training and genotyping, took about 12 hours (wall-clock time) on standard multi-core computer workstations with 16 Gbytes of memory.

Exome-sequencing data from Quartet A was used to assess the concordance of genotyping calls by QUADGT and a well-established variant caller, The Genome Analysis Toolkit [12]. We used independently produced whole-genome (WG) sequencing reads for the normal-tumor pair in Quartet B (with 124× total coverage, see Table 1) to gauge the two variant callers' sensitivity.

### Concordance experiments

Table 2 compares individual genotyping calls made by the Genome Analysis Toolkit [12] and QUADGT on a small example consisting of calls on chromosome 12 (parameters were set to result in a comparable number of genotyping calls). The table illustrates that most calls are made in agreement between the two programs. The known relationships between the samples ensure the consistency of calls made by QUADGT, resulting in only 4 putative *de novo *germline mutations. GATK, ignorant of the relations, has 327 cases where a normal allele does not appear at either parent, which is by at least two magnitudes higher than what one would expect based on human intergeneration mutation rates [6]. GATK genotypes imply a large number (520) of somatic mutations, as well. As with *de novo *mutations, the joint calls by QUADGT are more conservative: only 14 somatic mutation calls are made.

**Table 2:** Comparison of calls made by the Genome Analysis Toolkit and QUADGT on Quartet A.

Normal genome	
**Heterozygous SNPs **(ref/alt)	**Homozygous SNPs **(alt/alt)
called by both QUADGT and GATK: 2100	called by both QUADGT and GATK: 945
called by GATK only: 60	called by GATK only: 500
QUADGT calls ref/ref: 4	QUADGT calls ref/ref: 0
QUADGT calls alt/alt: 9	QUADGT calls ref/alt: 206
called by QUADGT only: 839	called by QUADGT only: 17
GATK calls alt/alt: 206	GATK calls ref/alt: 9
De novo mutations	
called by both QUADGT and GATK: 4	
called by GATK only: 327	
called by QUADGT only: 0	
**Tumor genome**	
**Heterozygous SNPs **(ref/alt)	**Homozygous SNPs **(alt/alt)
called by both QUADGT and GATK: 2032	called by both QUADGT and GATK: 938
called by GATK only: 41	called by GATK only: 504
QUADGT calls ref/ref: 3	QUADGT calls ref/ref: 0
QUADGT calls alt/alt: 7	QUADGT calls ref/alt: 224
called by QUADGT only: 989	called by QUADGT only: 17
GATK calls alt/alt: 224	GATK calls ref/alt: 7
**Somatic mutations**	
called by both QUADGT and GATK: 8	
called by GATK only: 512	
called by QUADGT only: 6	

### Sensitivity assessment

The normal-tumor pair in Quartet B was submitted to Illumina, Inc. for deep whole-genome (WG) sequencing and somatic mutation calling. Table 3 tallies the WG somatic calls with coverage by exome data. Based on the WG sequencing data, 1817 loci have somatic mutations, of which 40 are covered by exome reads to sufficient depth (Table 3, **b**). Some of the 40 WG somatic calls have no or weak support (lines **c **and **e**) in exome reads, since with at most one exception, all normal and tumor base calls are identical with the reference. The remaining 24 WG somatic calls (line **f**) with sufficient exome read coverage and non-reference base calls are not beyond the reach of the variant callers to discover somatic mutations. On closer inspection, 5 WG somatic calls fall into a 150 bp region (line **g**), which, judged by largely divergent base calls, is likely to be misaligned; 4 WG somatic calls (line **h**) may even be erroneous. For instance, at chr8:10078796, which has a fairly low WG somatic quality score, the parental exome reads suggest that all four samples contain a heterozygous SNP that is in fact found in dbSNP (rs112078536). The remaining 15 WG somatic calls (line **i**) have support in the exome reads, and QUADGT discovers them all at some quality threshold cutoffs.

**Table 3:** Whole-genome somatic loci and exome genotyping on Quartet B.

Locus	WG genotyping		**Exome base calls **(A:C:G:T)					Q**uadGT call**		
	**mutation**	**quality**	**N**	**T**	**F**	**M**	**N**	**T**	**F**	**M**

chr4:85818319	GG > AG	68	0:0:8:0	2:0:6:0	0:0:9:0	0:0:9:0	0/0	0/0	0/0	0/0
chr6:29965983	TT > CT	17	0:1:0:27	0:1:0:25	0:3:0:0	0:0:0:43	0/0	0/0	0/1	0/0
chr8:10078796	GG > CG	15	0:2:1:0	0:2:3:0	0:5:2:0	0:2:4:0	0/1	0/1	0/1	0/1
chr12:25289551	CC > TC	29	0:37:0:1	0:31:0:2	0:57:0:0	1:45:0:0	0/0	0/0	0/0	0/0

Genome coordinates refer to NCBI 36.1/hg18 genome build.

**a**. **WG somatic loci **in exome reads: 274

**b**. exome coverage ≥ 10: 40

**c**. only ref base calls in normal and tumor: 12

**d**. at least one non-ref base call in normal and tumor: 28 (= **c **- **d**)

**e**. exactly 1 non-ref base call in normal and tumor: 4

**f**. at least 2 non-ref base call in normal and tumor: 24 (= **e **- **f**)

**g**. within misaligned region chr19:4463156-4463205: 5

**h**. strong disagreement between exome and WG reads: 4

**i**. QUADGT calls SOMATIC: 15 (= **f **- (**g **+ **h**)) -- see Table 4

Table 4 compares the sensitivity of joint and independent genotyping using the 15 WG somatic calls with support in exome base calls (Table 3, line **i**). First, it is notable that QUADGT's tumor purity estimation (by Expectation-Maximization) is close to the Illumina's estimate from whole-genome data (see Table 1).

**Table 4:** Exome somatic calls supported by whole-genome data.

	Locus	Whole-genome						Exome			
		**mutation**	**score**	**Quality score**		**Rank**			**Base calls**	**(A:C:G:T)**	

				**qGT**	**GATK**	**qGT**	**GATK**	**Normal**	**Tumor**	**Father**	**Mother**

1	chr20:577556	GG > AG	61	282	99,99	1	1	0:0:90:0	27:0:64:0	1:0:104:0	1:0:90:0
2	chr18:26965596	AA > GA	87	282	99,99	1	1	77:0:1:2	63:1:20:0	106:0:0:0	84:0:0:0
3	chr9:139897201	GG > AG	59	91	66,99	16	73	0:0:23:0	6:0:18:0	0:0:24:0	0:0:21:0
4	chr2:88942555	GG > TG	93	53	-	84	-	0:0:29:0	0:0:5:3	0:0:36:0	0:0:26:0
5	chr2:88942554	AA > CA	93	52	-	86	-	30:0:0:0	0:0:6:3	0:0:36:0	0:0:26:0
6	chr7:150399212	CC > TC	93	48	60,99	98	104	0:20:0:0	1:6:0:9	0:14:0:0	0:13:0:0
7	chrX:129017457	CC > AC	41	46	45,99	109	221	0:15:0:0	5:6:0:0	0:16:0:0	0:12:0:0
8	chr16:3681082	CC > TC	89	44	30,82	118	629	0:10:0:0	0:10:0:4	0:19:0:0	0:11:0:0
9	chr10:107013067	CC > AC	30	36	-	186	-	0:52:0:0	5:57:0:1	0:74:0:0	0:72:1:0
10	chr8:72396806	TT > CT	59	24	-	398	-	0:0:0:6	0:3:0:13	0:0:0:20	0:0:0:11
11	chrX:21779651	GG > TG	89	21	36,99	483	412	2:0:13:0	0:0:7:8	0:0:9:0	0:0:14:0
12	chr5:125673987	CC > TC	97	14	6,70	790	7393	0:2:0:0	0:7:0:3	0:6:0:0	0:5:0:0
13	chr12:10156011	GG > AG	89	8	-	-	-	0:0:16:0	3:0:8:0	0:0:28:0	0:0:9:0
14	chr19:43602412	GG > AG	32	5	-	-	-	0:0:10:0	2:0:10:0	0:1:17:0	0:0:10:0
15	chr6:112619582	GG > AG	68	3	-	-	-	0:0:5:0	2:1:5:0	0:0:6:0	0:0:5:0

Somatic mutation calls of QUADGT (qGT columns) and the Genome Analysis Toolkit (GATK columns) were ranked by quality score (smaller of normal and tumor genotype call qualities in GATK's VCF output, and score of somatic call in QUADGT's VCF output that is *q *= - 10 log_10_(1 - *p*) for a mutation with posterior probability *p*).

Rank is "-" at loci where no somatic mutations were inferred by QUADGT or GATK. Genome coordinates refer to NCBI 36.1/hg18 genome build.

Table 4 suggests that the joint variant-calling in QUADGT leads to better sensitivity than GATK, which does not consider the relations between the genomes. In particular, 9 out of 276 (3%) SOMATIC calls by QUADGT with quality score at least 30 have support in the WG data, whereas only 7 of GATK's 667 (1%) divergent genotypes of same quality threshold are validated. At a quality score cutoff of 20, 12 out of 555 (2%) and 7 out of 1312 (0.5%) are validated QUADGT and GATK predictions.

## Conclusions

Sequencing multiple genomes with known pedigrees or clonal relationships has a great promise for understanding the development of particular diseases. Our experiments with sequenced quartets of parents and normal-tumor pairs illustrate that the joint calls improve the reliability of inferred *de novo *and somatic mutations. The constraints imposed by the known relationships greatly improve the consistency of calls between different samples, and ultimately help to delineate the single nucleotide polymorphisms that can be associated with the disease.

A future release of the software is now under development that incorporates more nuanced substitution models with variable rates, transition-transversion ratios and nucleotide composition, as well as site-specific priors relying on public variant databases and gene annotations.

## Competing interests

The authors declare that they have no competing interests.

## Declarations

Funding for the publication of this article was provided by a grant from the National Sciences and Engineering Research Council.

This article has been published as part of *BMC Bioinformatics *Volume 14 Supplement 5, 2013: Proceedings of the Third Annual RECOMB Satellite Workshop on Massively Parallel Sequencing (RECOMB-seq 2013). The full contents of the supplement are available online at http://www.biomedcentral.com/bmcbioinformatics/supplements/14/S5.
